# The predictive value of post-traumatic stress disorder symptoms for quality of life: a longitudinal study of physically injured victims of non-domestic violence

**DOI:** 10.1186/1477-7525-5-26

**Published:** 2007-05-21

**Authors:** Venke A Johansen, Astrid K Wahl, Dag Erik Eilertsen, Lars Weisaeth, Berit R Hanestad

**Affiliations:** 1 Faculty of Health Buskerud University College, Konggt 51, 3019 Drammen, Norway; 2 Resource Centre on Violence, Traumatic Stress and Suicide Prevention, Western Norway (RVTS-West), Ulriksdalen 2, Helse Bergen, Haukeland University Hospital, Norway; 3 Institute of Nursing and Health Sciences Medical Faculty, University of Oslo, Pb 1153 Blindern, 0316 Oslo, Norway; 4 Department of Psychology, University of Oslo, Pb 1094 Blindern, 0317 Oslo, Norway; 5 Norwegian Centre for Violence and Traumatic Stress Studies (NKVTS), Ullevål University Hospital, Kirkeveien 116, 0407 Oslo, Norway; 6 Department of Public Health and Primary Health Care, University of Bergen, Kalfarveien 31, 5018 Bergen, Norway

## Abstract

**Background:**

Little is known about longitudinal associations between post-traumatic stress disorder (PTSD) and quality of life (QoL) after exposure to violence. The aims of the current study were to examine quality of life (QoL) and the predictive value of post-traumatic stress disorder (PTSD) for QoL in victims of non-domestic violence over a period of 12 months.

**Methods:**

A single-group (n = 70) longitudinal design with three repeated measures over a period of 12 months were used. Posttraumatic psychological symptoms were assessed by using the Impact of Event Scale, a 15-item self-rating questionnaire comprising two subscales (intrusion and avoidance) as a screening instrument for PTSD. The questionnaire WHOQOL-Bref was used to assess QoL. The WHOQOL-BREF instrument comprises 26 items, which measure the following broad domains: physical health, psychological health, social relationships, and environment. Results of the analysis were summarized by fitting Structural Equation Modelling (SEM).

**Results:**

For each category of PTSD (probable cases, risk level cases and no cases), the mean levels of the WHOQOL-Bref subscales (the four domains and the two single items) were stable across time of assessment. Individuals who scored as probable PTSD or as risk level cases had significantly lower scores on the QoL domains such as physical health, psychological health, social relationships and environmental than those without PTSD symptoms. In addition, the two items examining perception of overall quality of life and perception of overall health in WHOQOL showed the same results according to PTSD symptoms such as QoL domains. PTSD symptoms predicted lower QoL at all three assessments. Similarly PTSD symptoms at T1 predicted lower QoL at T2 and PTSD symptoms at T2 predicted lower QoL at T3.

**Conclusion:**

The presence of PTSD symptoms predicted lower QoL, both from an acute and prolonged perspective, in victims of non-domestic violence. Focusing on the individual's perception of his/her QoL in addition to the illness may increase the treatment priorities and efforts.

## Background

The human response to interpersonal violence, is one of the most important public health problems in the world [1]. Exposure to a terrifying event such as violence may confront an individual with such horror and threat to a degree that usual psychological defenses are incapable of coping with the impact. The consequences may be temporarily or permanently altered capacity to cope, changed concept of self and reduced quality of Life (QoL). Research shows that the anxiety disorder, post-traumatic stress disorder (PTSD) is a common problem following violence, and that other emotional problems may be secondary to PTSD [2,3].

Three clusters of symptoms, namely re-experiencing, avoidance and hyperarousal define PTSD. In almost all persons, intrusive and repetitious symptoms develop after exposure to extreme stress. However, only a certain proportion develop avoidance and hyperarousal symptoms [4]. The risk of posttraumatic emotional problems has been found to be highest in persons who report that during the assault they feared they would be killed or seriously injured, or actually were injured [2,5]. Prior experiences of victimization have also been found to elevate the risk of emotional problems following new victimization [6]. In other studies, experiences of earlier violence, perceived threat and injury severity have been found to be important predictors of PTSD [2]. Individuals who develop symptoms of PTSD usually recover within one year after the event. Those who do not rarely recover completely [7].

Knowledge about people's experience of reactions following exposure to violence, including the impact on their QoL, is needed to improve the understanding of these complex psychological processes [8]. Publications on the subject of QoL in psychiatric research are of later date than those in somatic medicine [9]. Quality of Life (QoL) has been defined in a number of ways such as symptom status, functional health, general health perceptions, general life satisfaction, well-being and overall QoL. Terms such as health-related QoL, functional status, subjective health status and overall QoL are used interchangeably to express different aspects of the term QoL in the field. Numerous questionnaires have been developed for assessing the construct. Most authors agree that QoL should be approached as a complex and multidimensional construct [10,11]. The World Health Organization defines QoL as: "the individual's perception of his/her position in life in the context of the culture and value system in which he/she lives and in relation to his/her goals, expectations, standards and concerns" [12]. This definition reflects the multidimensional nature of QoL as the subjective evaluation is embedded in the individual's physical health, psychological state, level of independence, social relationships, personal beliefs and relationships to salient features of the environment [12].

The relationship between physical symptoms, health status, psychological status and satisfaction with life is complex [13,14]. Wilson and Cleary (1995) constructed a conceptual model of health-related quality of life (HRQoL) that integrates both biological and psychological aspects of health outcomes linked with both individual and environmental characteristics [15]. This model linked physiological variables, symptom status, functional health, general health perceptions and overall QoL. Health perception, subjective measures of life satisfaction and well-being are not found directly as a one-to-one relationship to severity of symptoms, disability and functional limitations in their review of research on interrelationships of patients' outcome [15]. The model integrates a continuum of increasing levels of complexity for understanding the impact on QoL. The causal pathway of the model begins with biological aspects where overall QoL is the final outcome. The model has been widely applied to examine populations with a spectre of different diseases according to QoL [16].

The European Study of Epidemiology of Mental Disorders (ESEMeD) reported that mental disorders were associated with substantial levels of disability and loss of QoL [17]. Some QoL assessments reflect a new evaluation of functional and social outcomes associated with recovery from mental illness. The assessments of QoL in the psychiatric field are emerging as important, both in consideration of different diagnoses and in consideration of the impact of treatment intervention, and also in evaluation of medical disability.

Several studies of Vietnam veterans examining the impact of PTSD on QoL by a wide range of QoL measures, show that PTSD have negative influence on QoL in both females and males [18-20]. The influence on QoL is not found only among the veterans with the diagnosis of PTSD, but also among family members [21]. Still there is an obvious lack of research on the implications of PTSD for QoL [10,11,22,23]. Also QoL studies based on civilian populations have been shown to predict QoL impairment in patients diagnosed as suffering from PTSD [10,11,22,23].

How PTSD- symptoms after exposure to non-domestic violence influence QoL is less known, as well the impact of PTSD on QoL over time. As far as we know, no longitudinal studies of civilians have evaluated the relationship between QoL and PTSD after exposure to non-domestic violence. The aims of the present study are as follows.

1) To investigate QoL in victims of non-domestic violence by assessing the appearance of PTSD symptoms over a one-year period following the trauma.

2) To investigate the predictive value of prior experience of violence, level of physical injury, perceived life threat and the presence of PTSD symptoms on QoL in victims of non-domestic violence over a one-year period following the trauma.

## Methods

### Design

The present study is a part of a larger study of the consequences' of non-domestic violence, combining semi-structured interviews and questionnaires. This study had a single-group (n = 70) longitudinal design with three repeated measures over a period of 12 months. Most respondents (97%) answered the first questionnaire during a period that ranged from a few days to 16 weeks after the assault (T1). The second assessment was conducted 3 months later (T2) and the third assessment was 12 months later than the first assessment (T3).

### Sample and data collection

The criteria for inclusion were people aged 18 years or older seeking assistance from an emergency unit or making a police report of actual physical assault in the communities of Bergen and Oslo, Norway. For inclusion the person had to be assaulted by a person other than a family member or a present or former intimate partner. With the assistance of local police and medical services, participants were identified and recruited. Following ethics committee approval, potential participants were asked permission for the researcher to contact them. If the person agreed, informed consent and more information about the project were sent by post.

**Figure 1:**
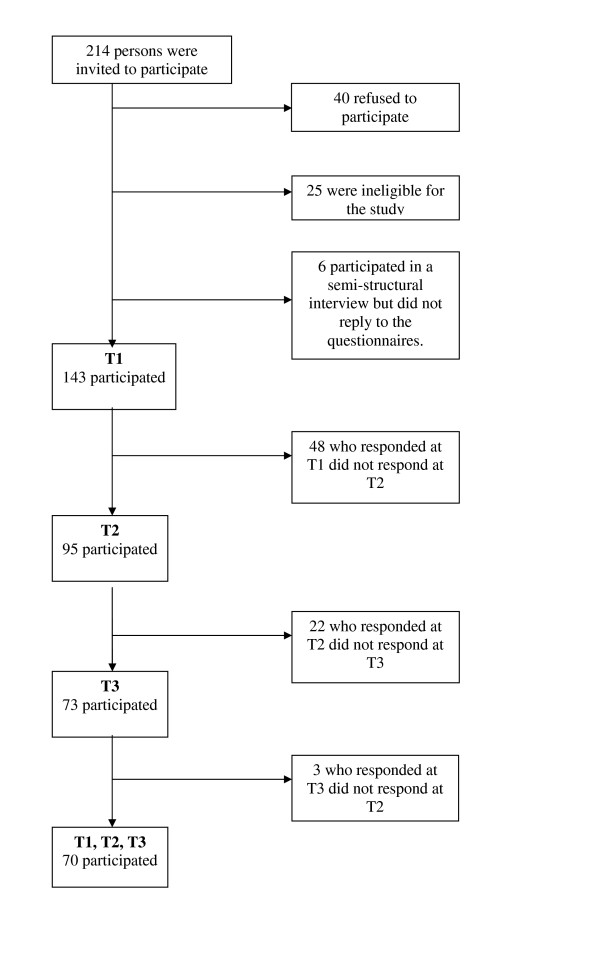
Flow chart: Recruitment.

The flow chart in Figure 1 shows that 214 people were asked to participate. Forty refused; this group had an average age of 29.6 (range 18–66) years and gender distribution of 37 men and 3 women. Twenty-five people were ineligible for the study because they failed to satisfy the criteria for study entry. Six persons participated in a semi-structured interview but did not return the questionnaires.

The sample at first assessment (T1) therefore consisted of 143 Norwegian-speaking adults. The response rate was 66% (n = 95) at T2 and 51% (n = 73) at T3. Fourteen could not be reached by mail at T3 due to their addresses being unknown.

Seventy persons (49%) participated at all three assessments. The average age in the respondent group at all three assessments was 33 years (SD = 12.3) with a range from 18 to 75 years, and the gender distribution was 83% (n = 58) male and 17% (n = 12) female participants. The 70 respondents who participated at all three assessments had all been physically injured during the assault.

Table 1 presents information on all participants at T1, persons who participated at all three assessments ("respondents") and dropouts.

**Table 1:** Sample characteristics.

	*Participants at T1*	*Respondents at T1, T2 and T3*	*Dropouts*
*Sample size*	143	70	73

*Age*			
*Mean (SD)*	31 (11.0)	33 (12.3)	28 (9.3)
*Range*	18–75	18–75	18–57
			
*Gender *% (n)			
Male	80% (114)	83% (58)	77% (56)
Female	20% (29)	17% (12)	23% (17)
			
*Prior experience of violence *% (n)			
Yes	48% (63)	45% (29)	51% (34)
No	52% (69)	55% (36)	49% (33)
			
*Physical injury *% (n)			
Assault	31% (45)	30% (21)	33% (24)
Inflicted bodily harm	69% (98)	70% (49)	67% (49)
			
*Cohabitation *% (n)			
Living with others	60% (86)	58% (41)	61% (45)
Living alone	40% (57)	42% (29)	39% (28)
			
*Marital status *% (n)			
Single	71% (101)	69% (48)	74% (53)
Married/cohabitant	18% (25)	19% (13)	17% (12)
Separated/divorced	11% (16)	12% (9)	10% (7)
			
*Educational level *% (n)			
Primary school	8% (11)	6% (4)	10% (7)
Secondary school	56% (81)	47% (33)	67% (48)
University, less than 4 y.	27% (38)	34% (24)	19% (14)
University more than 4 y.	9% (12)	13% (9)	4% (3)
			
*Employment *% (n) *			
Employed/self-employed	66% (95)	67% (47)	65% (48)
Students/military service	24% (35)	26% (18)	23% (17)
Unemployed/grant leaved	11% (16)	7% (5)	15% (11)
Pensioned/sick leaved	9% (13)	13% (9)	5% (4)
			
*Threat level *% (n)			
Felt life at risk	41% (50)	41% (25)	41% (25)
Fear of severe physical injury	21% (25)	21% (13)	19% (12)
Understood danger afterwards	12% (15)	13% (8)	12% (7)
Did not perceive dangerous	23% (28)	23% (14)	23% (14)
Did not remember	3% (4)	2% (1)	5% (3)

* Employment: The total is more than 100% as some participants were both employed and studying or both employed and pensioned

Independent t-test showed a statistically significant difference in mean age between respondents and dropouts (t = 2.57, p = 0.01, df = 128), with respondents an average of five years older than dropouts. Similarly, independent t-test showed statistically significant differences in mean educational level in respondents and dropouts (t = 2.25, p = 0.03, df = 135), where respondents had a higher level of educational than dropouts. No statistically significant differences were found between respondents and dropouts with regard to gender, prior experience of violence, level of physical injury, cohabitation, marital status, employment status or threat level. Further, there were no statistically significant differences between respondents and dropouts with regard to mean values on scales and subscales of IES-15 and WHOQOL-Bref.

### Assessment

#### Quality of life

The WHOQOL-Bref is a self-report scale that consists of 26 items. It is a multilingual, multicultural generic quality of life scale, developed across 15 field centres [12,24]. The WHOQOL-Bref includes four domains related to QoL: physical health, psychological health, social relationships and environment. In addition, two items are examined separately, namely the perception of overall quality of life and perception of overall health. The WHOQOL-Bref has been demonstrated to have satisfactory discriminant validity, internal consistency and test-retest reliability [12,25]. The Norwegian version used in the present study has also been reported to have satisfactory psychometric properties [26]. The items are rated on a 5-point Likert scale, reflecting intensity, capacity, frequency or evaluation. The items inquire "how much", "how completely", "how often", "how good" or "how satisfied", with possible answers ranging, from very satisfied [5] to not at all satisfied [1]. The range of scores in each domain is from 4 to 20, where a higher score indicates a better QoL. In the present study, all measurement domains show satisfactory internal consistency and reliability, as estimated by Cronbach's alpha: physical health = 0.87, psychological health = 0.84, social relationships = 0.88 and environment = 0.87.

#### Post-traumatic stress disorder symptoms

The Impact of Event Scale-15 (IES-15) has been demonstrated to be a useful self-report measure of stress reactions after the experience of a traumatic event, and to be valuable for detecting individuals who need treatment [27-29]. The items are scored on a 4-point scale, scored as 0 (not at all), 1 (rarely), 3 (sometimes) and 5 (often). In research, the intrusion and avoidance subscales from the IES-15 are typically used. Scores range from 0 to 35 for intrusion, 0 to 40 for avoidance and 0 to 75 for the total IES-15. On the full scale, a total score of 35 or more has been reported to indicate PTSD, and a score between 20 and 34 indicates a level of risk [30]. In the present study, internal consistency as assessed by Cronbach's alpha was found to be: IES-15 total = 0.83, intrusion subscale = 0.96 and the avoidance subscale = 0.96.

#### Perception of life threat

The victims' perception of threat to life and their fear of increased severe physical injury were categorized as: felt life at risk, fear of increased severe physical injury (but life not at risk), understood danger afterwards, did not perceive the situation as dangerous, and did not remember.

#### Classification of physical injury

The participants were recruited from the two main crime categories used by the police in their registration of violence: "assault" and "inflicted bodily harm" [31]. Each case was classified at T1 in cooperation with the police, based upon a judgement made using a combination of the level of physical injury and severity of intention of the perpetrator to cause harm, where physical injury is the most important criterion. The assault category comprises injuries ranging from a black eye to those that are quite serious, and in addition often includes serious threats of more severe physical injury. The victims of inflicted bodily harm comprise people with more serious physical injuries ranging from near fatal injuries to different kinds of fractures, or other comprehensive bodily injuries.

#### Previous experience of being a victim

Previous experience of being a victim were categorised as yes or no.

#### Demographics

Demographic information such as age, gender, educational level, cohabitation, marital status and employment status were recorded.

### Statistical analysis

**Figure 2:**
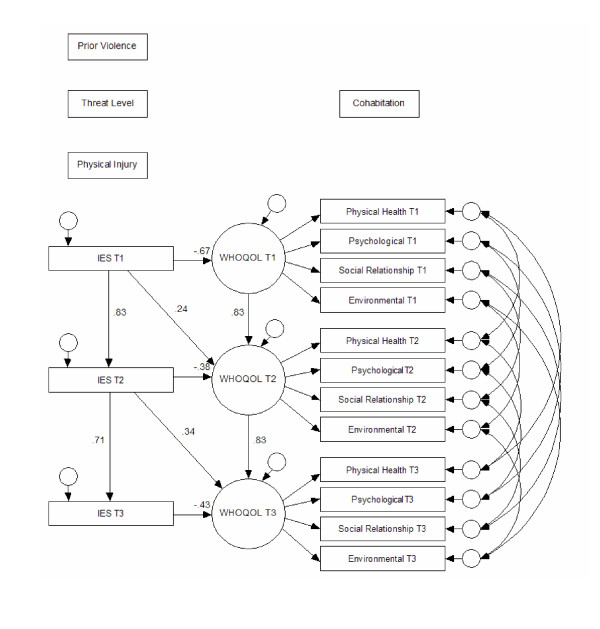
Structural equations model fitted to the data. IES T1, IES T2 and IES T3 = Impact of Event Scale-15 at T1, T2 and T3, WHOQOL T1, WHOQOL T2 and WHOQOL T3 = WHOQOL-Bref at T1, T2 and T3, Prior violence = Previous experience of being a victim of violence, Threat level = The victims' perception of threat, Physical injury = severity of physical injury categorized as "assault" or "inflicted bodily harm", cohabitation = living alone or living with others

Data were analysed by frequency tabulations, cross tabulations, independent sample t-tests, Pearson's *r *and analysis of variance. Results of the analyses were summarized by fitting Structural Equation Modelling (SEM) to data of persons who participated at all three assessments. The construction of the aims and analysis including variables such as prior violence, threat level, and physical injury in figure 2 is based on earlier findings, for instance prior SEM-analyses examining predictors of PTSD in a cross sectional perspective at T1 [2] and a longitudinal perspective including all the 3 measurement [32]. The arrows in the SEM-model represent the hypothesized linkages between the dimensions already analysed and the pathways presented in Wilson and Cleary conceptual model [15]. Cohabitation is believed to influence health and perception of QoL [33,34]. All analyses were performed using SPSS v.14 and AMOS v.6.

## Results

### Sample characteristics

Table 1 shows that the sample participating at all three assessments comprised 83% male and 17% female victims with an average age at 33 years (*SD *= 12.3, range 18–75). Thirty percent of the participants were categorized as "assault" and 70% as "inflicted bodily harm" according to physical injury. Forty-one percent felt that their life was at risk during the assault and 21% felt that they were in danger and could obtain severe injuries, but did not feel that their life was at risk. For further information about sample characteristics see table 1.

### Scale scores and the level of QoL by probability of PTSD

Descriptive information on the scales and subscales for respondents at T1, T2 and T3 is shown in Table 2.

**Table 2:** Descriptive information on scales and subscales for those who participated at all three assessments (n = 70).

		**T1**		**T2**		**T3**	
Scale	Subscales	Mean	SD	Mean	SD	Mean	SD

*IES-15*	Total	26.0	18.4	23.1	17.1	22.1	19.5
	Intrusion	13.6	10.1	11.3	9.2	10.4	9.5
	Avoidance	12.3	10.5	9.4	11.8	12.0	11.8
*WHOQOL- Bref*	Physical health	14.9	3.6	15.4	3.2	15.1	3.4
	Psychological	13.9	3.1	14.4	3.0	14.1	3.4
	Social relationships	14.7	3.2	14.3	3.4	14.8	3.8
	Environmental	14.5	2.7	14.9	2.7	14.7	3.0
	Overall QOL	3.6	1.0	3.7	0.8	3.6	1.0
	Overall Health	3.4	1.1	3.4	1.0	3.5	1.0

The respondents were classified as probable PTSD cases, risk level PTSD cases and no PTSD cases, according to scores on the IES-15. Table 3 shows scores on the WHOQOL-Bref (overall QoL, overall health and the four sub-domains) by probability of full or partial PTSD at T1, T2 and T3.

**Table 3:** Mean scores on WHOQOL-Bref by probability of PTSD for respondents at T1, T2 and T3 (n = 70).

Time	**T1**			**T2**			**T3**		
Probability of PTSD	Probable PTSD	Risk level	No cases	Probable PTSD	Risk level	No cases	Probable PTSD	Risk level	No cases

	Mean (SD)	Mean (SD)	Mean (SD)	Mean (SD)	Mean (SD)	Mean (SD)	Mean (SD)	Mean (SD)	Mean (SD)

*Overall QoL*	2.94 (0.94)	3.52 (0.99)	4.14 (0.69)	3.14 (0.85)	3.80 (0.62)	4.03 (0.78)	2.86 (0.89)	3.50 (0.85)	4.10 (0.73)
*Overall Health*	2.94 (1.26)	3.00 (1.00)	4.03 (0.73)	2.95 (1.07)	3.25 (1.11)	3.72 (0.75)	2.61 (0.92)	3.60 (1.07)	3.87 (0.78)
*Physical health*	12.03 (3.77)	13.79 (2.86)	17.45 (1.81)	12.91 (3.27)	14.77 (2.63)	17.64 (1.67)	12.23 (3.41)	14.86 (2.56)	16.84 (2.18)
*Psychological*	11.89 (2.90)	12.69 (2.28)	16.14 (2.41)	12.14 (2.45)	14.40 (2.66)	16.14 (2.47)	11.54 (3.11)	13.20 (2.86)	15.89 (2.63)
*Social*	12.89 (3.03)	14.03 (2.75)	16.32 (2.89)	12.06 (3.17)	14.66 (3.32)	15.63 (2.85)	12.60 (3.92)	14.26 (3.97)	16.07 (3.20)
*Environmental*	12.72 (2.97)	13.58 (1.77)	16.41 (1.77)	13.17 (2.37)	14.21 (2.77)	16.61 (1.64)	12.67 (3.11)	14.25 (2.73)	16.05 (2.36)
*Number of respondents*	18 (26%)	23 (33%)	29 (41%)	21 (30%)	19 (29%)	29 (41%)	22 (31%)	10 (14%)	38 (54%)

Probable PTSD is "diagnosed" if IES-15 scores are > = 35, risk level if scores are > = 20 and < 35 and no PTSD if scores < 20.

Generally, WHOQOL-Bref values associated with probable PTSD were lower than values associated with no cases, for instance, at T1: mean level of physical health was 12.03 for those diagnosed as probable PTSD, while the corresponding value was 17.45 for those classified as no cases. One-way ANOVAs showed statistically significant main effects of the probability of PTSD for all WHOQOL-Bref subscales at all three assessments. With the exception of overall health at T2, where p < 0.05, all other p values were < 0.001.

For each category of PTSD (probable cases, risk level cases and no cases), the mean levels of the WHOQOL-Bref subscales (the four domains and the two single items) were stable across time of assessment: for instance, the mean scores for the domain "psychological health" at T1 was 11.89, while the corresponding means at T2 and T3 were 12.14 and 11.54, respectively.

### QoL predicted by prior experience of violence, level of physical injury, perceived life threat and presence of PTSD symptoms

Table 4 shows bivariate correlations among IES-15 and WHOQOL-Bref scales and subscales at T1, T2 and T3. All correlations (ranging from 0.29 to 0.87) were statistically significant (p < .01).

**Table 4:** Pearson's correlation among measures of PTSD (IES-15) and QoL (WHOQOL-Bref) by all three times of assessment (n = 70).

		**T1**						**T2**							**T3**						
		2	3	4	5	6	7	1	2	3	4	5	6	7	1	2	3	4	5	6	7

**T1**	1 IES-15	-.66	-.57	-.45	-.53	-.54	-.43	.83	-.61	-.54	-.44	-.51	-.42	-.37	.65	-.38	-.43	-.32	-.36	-.39	-.36
	2 Physical health	1	.72	.63	.71	.74	.68	-.62	.77	.69	.59	.60	.50	.62	-.45	.59	.58	.56	.56	.57	.43
	3 Psychological		1	.72	.74	.76	.59	-.56	.64	.81	.70	.68	.60	.54	-.40	.50	.68	.65	.59	.54	.43
	4 Social relationships			1	.63	.66	.52	-.41	.57	.69	.76	.58	.43	.47	-.29	.45	.63	.72	.55	.54	.34
	5 Environmental				1	.70	.62	-.52	.61	.61	.66	.81	.49	.51	-.32	.53	.58	.61	.71	.53	.44
	6 Overall QoL					1	.66	-.47	.61	.64	.73	.65	.69	.59	-.37	.51	.59	.56	.56	.54	.49
	7 Overall health						1	-.36	.70	.60	.61	.63	.53	.81	-.35	.55	.53	.52	.60	.61	65

**T2**	1 IES-15							1	-.67	-.59	-.49	-.57	-.48	-.33	.71	-.51	-.53	-.34	-.44	-.44	-.32
	2 Physical health								1	.78	.72	.72	.66	.65	-.54	.74	.71	.64	.62	.67	.61
	3 Psychological									1	.70	.69	.65	.54	-.45	.57	.73	.66	.57	.65	.61
	4 Social relationships										1	.73	.72	.53	-.39	.58	.70	.74	.57	.65	.49
	5 Environmental											1	.65	.48	-.40	.62	.66	.61	.76	.68	.48
	6 Overall QoL												1	.56	-.52	.62	.66	.53	.47	.67	.56
	7 Overall health													1	-.36	.50	.46	.39	.45	.53	.69

**T3**	1 IES-15														1	-.61	-.60	-.41	-.46	-.60	-.53
	2 Physical health															1	.87	.67	.76	.79	.75
	3 Psychological																1	.79	.78	.83	.66
	4 Social relationships																	1	.68	.76	.52
	5 Environmental																		1	.72	.56
	6 Overall QoL																			1	.63

Correlations (r) > 0.40 are significant at 0.001 level, 0.40 > r > 0.29 are significant at 0.01 level and r < 0.29 are significant at 0.05 level.

Table 5 shows bivariate correlations among IES-15, WHOQOL-Bref total scores and sample characteristics at T1, T2 and T3. IES at T1 (p < 0.05) and QoL at T1, T2 and T3 were significantly correlated with age (p < 0.05).

**Table 5:** Pearson's correlation among sample characteristics and measures of PTSD (IES-15, totalscore), QoL (WHOQOL-Bref-totalscore) by all three times ofAssessment (n = 70).

	Age	Prior violence	Physical injury	Threat level	Cohabitation
IES-T1	0.26*	0.11	0.03	0.14	-0.03
IES-T2	0.16	0.04	-0.07	0.09	-0.06
IES-T3	0.33	0.08	-0.08	0.03	-0.22
QoL-T1	-0.26*	-0.41	-0.07	-0.25	-0.02
QoL-T2	-0.27*	-0.03	-0.02	-0.24	0.12
QoL-T3	-0.30*	-0.06	-0.04	-0.24	0.10

*p < 0.05

The SEM analysis shown in Figure 2 summarizes the statistically significant relations among all relevant variables, including variables such as prior violence, threat level, physical injury, cohabitation, IES-15 and WHOQOL-Bref (QoL). While the main purpose of the SEM analysis is to summarize the relations among variables in the study, the model is obviously also based on a theoretical understanding of the relation between PTSD symptoms and quality of life [15] and prior research [10,18-21,35-37]. The four domains of physical health, psychological, social relationships and environment were modelled as indicators of a common component. Regression coefficients (b), standard errors (S.E.), critical ratios (C.R.), standardized regression values (beta), and p-values are presented in table 6. *R*-square was 0.69 for IES-15 at T2, 0.51 for IES-15 at T3, 0.45 for WHOQOL-T1, 0.82 for WHOQOL-T2 and 0.75 for WHOQOL-T3. The model with 138 degrees of freedom fitted the data reasonably well (RMSEA = 0.065), chi-square/df = 1.3. Arrows between variables indicate statistically significant effects. Two-way arrows show correlations between error terms for variables measured repeatedly at T1, T2 and T3.

**Table 6:** Regression coefficients (b), standard errors (S.E.), critical ratios (C.R.), p-values (p) and standardized regression coefficients (beta) from SE model fitted to data (see figure 2).

	b	S.E.	C.R.	P	beta
IES-T1 → QoL-T1	-1.758	0.275	-6.389	< 0.001	-0.673
IES-T1 → IES-T2	0.770	0.062	12.488	< 0.001	0.833
IES-T2 → IES-T3	0.815	0.096	8.464	< 0.001	0.714
IES-T2 → QoL-T2	-0.971	0.282	-3.446	< 0.001	-0.384
IES-T1 → QoL-T2	0.569	0.295	1.928	0.054	0.243
Indirect effect of IES-T1 on QoL-T2	-2.047				-0.877
QoL-T1 → QoL-T2	0.739	0.092	8.057	< 0.001	0.827
IES-T3 → QoL-T3	-0.994	0.225	-4.426	< 0.001	-0.427
IES-T2 → QoL-T3	0.906	0.303	2.991	0.003	0.341
QoL-T2 → QoL-T3	0.878	0.111	7.937	< 0.001	0.835
Indirect effect of IES-T2 on QoL-T3	-1.662				-0.626

Scores on IES-15 predicted QoL at all three assessments. IES scores at T1 predicted QoL at both T1 (p < 0.001) and at T2 (p = 0.05). Similarly, IES scores at T2 predicted QoL at T2 (p < 0.001) and T3 (p < 0.01). QoL at T1 was found to be a predictor of QoL at T2, and QoL at T2 predicted QoL at T3 (all p < 0.001). The effects of IES-T1 on QoL-T2, and IES-T2 on QoL-T3, were expected to be negative, but turned out to be positive.

Missing arrows between variables in the path diagram indicate that these effects were not statistically significant and they were constrained to zero in the final model. Experiences of earlier violence, perceived threat, severity of injury or cohabitation (living alone or living together with others), were not significant predictors of QoL.

To further explore the relationships among IES and QoL, a modified SE model were fitted to data. The alternative model was a more complete "cross-lagged" model, estimating the direct effects of both IES on QoL and of QoL on IES. Table 7 shows standardized regression coefficients (beta), p-values and RMSEA for the two different SEMs.

**Table 7:** Standardized regression coefficients (beta), p-values and rmsea for two SEModels

	*SEmodel 1*		*SEmodel 2*	
	beta	p	beta	p

IES-T1 → QoL-T2	0.24	0.054	0.23	0.051
IES-T2 → QoL-T3	0.34	0.003	0.33	0.003
QoL-T1 → IES-T2	0	-	-0.16	0.102
QoL-T2 → IES-T3	0	-	-0.07	0.586
RMSEA	0.065		0.065	

The model fits of the two models were identical (RMSEA = 0.065). In the alternative model, the effects of IES on QoL were unchanged from the first model, and the direct effects of QoL on IES were weak and failed to reach statistical significance. The first model is a convenient way of summarising the correlation pattern among the observed variables and the in-direct effects of IES on QoL is in line with out theoretical understanding of the relationship between IES and QoL.

## Discussion

### The level of QoL by probability of PTSD

Our results showed lower mean values of the four domains (physical health, psychological health, social relationships, environment) and the two items (overall QoL and overall health) of the WHOQOL-Bref, in those suffering from probable PTSD compared to those diagnosed as no cases at all times of assessment.

The negative impact of PTSD on QoL is evident in our results, and in accordance with earlier findings based on a wider range of QoL measurements in both veteran and civilian populations [10,18-21,35-37]. Respondents in the present study categorized as probable cases or risk cases also had lower QoL in all four domains and the two single items, than participants in a study of the Norwegian general population [26]. Our results also showed that respondents categorized as no cases had a similar or even better QoL than participants in this Norwegian study [26]. Result of the present study are in accordance with other research findings, for instance Warshaw et al (1993) found worse QoL functioning among patients diagnosed with PTSD than among patients without the experience of potentially traumatic events [37]. Schnurr et al (2006) in their study of veterans found that PTSD symptoms were associated with reduced health related QoL [18]. They found consistent results across psychosocial and physical domains, but with stronger effect in the psychosocial domain. Our results also are similar to findings of Rapaport et al (2005) which showed that 59% of PTSD patients and 63% of the patients with major depression had severe QoL impairment [10].

One study that examined the presence of PTSD and QoL as outcome measures in a small sample of clients in a community mental health setting, using the WHOQOL-Bref, reported a significant reduction of QoL in all domains [23]. Another study, which intended to validate the Swedish Quality of Life Inventory (QOLI), used the questionnaire in a group of crime victims who suffered from PTSD. They reported significantly lower QoL in the PTSD group than in a matched non-clinical group, with large differences in the life areas of self-regard, love relationships, creativity, learning, standard of living, work, health, philosophy of life, recreation, community and friendship [38]. All these studies included the present study, and the pathway pointed out in the model of Wilson and Cleary [15] suggest that, independent of the QoL questionnaire used for measurements, there is an association between PTSD and reduced QoL.

Our results are in accordance of most psychiatric studies investigating the relationship between subjective QoL and psychopathology in terms of psychiatric symptoms [39]. The areas of depression and anxiety have especially been pointed out regarding this relationship [39]. In that point of view our results are expected, PTSD is categorised as anxiety disorder with high comorbidity with anxiety and depression.

Assessment of QoL after exposure to non-domestic violence will give an evaluation of the persons' subjective perception of quality of his or hers own life [10,11], and would be valuable in determining information beyond the symptoms of PTSD, such as the impact of treatment [10,40] and in order to evaluate medical disability.

### The predictive value of PTSD symptoms for QoL

The present study showed that PTSD symptoms may predict reduced QoL at all times of measurement. Figure 2, which summarizes the results, identifies PTSD symptoms as defined by high IES-15 scores, as a predictor of reduced QoL at all three assessment times. PTSD symptoms were found to be a predictor of lowered QoL, both when measured concurrently and when measured at all prior assessments. Our study showed high correlations, high explained variance and statistically significant results, which all support the conclusion of probable PTSD as an important predictor of poor QoL.

Similar conclusions are also relevant to draw based on the results of the alternative SEM analysis fitted as a more "complete" "cross-lagged" model. Our findings of the effects of PTSD-symptoms on QoL to be unchanged from the first model and no statistical significant of the effects of QoL on PTSD-symptoms indicates that the correct pathway arrow is from to PTSD-symtoms to QoL. Our results indicate that PTSD symptoms are important and powerful factors that negatively influence the person's experience of QoL, in accordance with several other studies showing PTSD with negative influence on QoL [10,18-21,35-37].

In previous a paper presenting results from a cross-sectional analysis of acute psychological reactions of 138 non-domestic victims of violence [3], we found perceived threat to be a predictor of peritraumatic dissociation, and peritraumatic dissociation to be a predictor of PTSD at T1. Our results showed that perceived life threat or fear of severe physical injury during the event was a direct predictor of PD, but not a predictor of PTSD. A longitudinal analysis likewise identifies perceived threat as an underlying predictor of PD, and PD as a predictor of PTSD after being exposed to violence presented in another paper [32].

Preliminary evidence suggests that PTSD and panic disorder may have a stronger influence on perceived QoL than other anxiety disorders [11]. A longitudinal study investigating the relationship between PTSD and health related QoL in injured trauma victims over a period of 12 months found PTSD to be a predictor of reduced QoL [35]. Injury was intentional for 15% of their sample. Another longitudinal study examining the influence of PTSD on QoL at 6-, 12- and 18 months of follow-up after exposure to major trauma (several trauma types) also reported high impact of PTSD on QoL [36].

In the SEM-analyses the direct effects of IES-T1 on QoL-T2 and IES-T2 on QoL-T3 showed up as positive numbers. This was not an expected result because of the inverse direction of the scales. The overall effects of IES-T1 on QoL-T2 as well as of IES-T2 on QoL-T3 measured by the bivariate correlation coefficients showed as expected up as negative numbers (se table 4). This inverse result is difficult to explain, but some hypotheses may be suggested. One possible explanation may be the "Time Principle of Re-appraisal", finding that dissatisfaction caused by a significant negative event decreases over time from [41]. Another alternative may be "the Principle of What Might Have Been of Re-evaluation", understood as comparing negative events in own life with fictitious occurrence what might have been worse, with the result of decreased dissatisfaction of a life domain. To fully understand this seemingly contradictory effect, further research will be necessary.

Experiences of earlier violence, perceived threat or injury severity were not found as predictors of QoL in the present study. Research shows that living in a partnership is an important determinant of psychological and social well-being in depressed individuals [33], and that poor family support may influence more dysfunctional coping styles [42]. While cohabitation (living alone or living together with others) was expected to be a predictor of QoL, the results showed no significant connection in our study.

### QoL-studies in the psychiatric field

The constructs of PTSD, psychological, physical health and QoL are probably closely related but believed to be distinct, such as the construct of depression related to these other concepts [43]. Research has shown that subjective QoL is particularly poor in depressed populations [40,44]. Doubts have been raised that subjective QoL measures may be contaminated by psychopathological symptoms, especially considering depression symptoms.

For instance, such comments were made in a study that evaluated depressive symptoms and QoL outcomes using the WHOQOL-Bref [44]. Because of high correlations in our study between values obtained from the WHOQOL-Bref (the four domains) and those from the IES-15, it may be reasonable to assume that assessing QoL in individuals with PTSD symptoms may be tautological measures. However, comparing the questionnaires IES-15 and WHOQOL-Bref showed that only one single item, sleep quality, focused on a similar area. Therefore, the high correlation may not be due to a measurement overlap. To further address this issue, we evaluated the relationship between the overall QoL item and IES-15 scores. These results also showed both high correlations and explained variance, and supported the conclusion of probable PTSD as a powerful predictor of poor QoL.

Priebe et al (1999) points out that basically psychopathology and QoL are independent constructs, but high association between their relationships deserves further research and attention [39]. They have in mind that longitudinal research with repetitious assessment design will throw more light on causality and reciprocal interaction over time than most of studies with cross-sectional designs. Another aspect of importance is that the individual evaluation of his/her own life through self reported QoL is quite different from measurement of symptoms through IES-15. The two questionnaires represent independent aspects of people's experience and functions. Indicating areas such as social relationships, environment and the two single items, the WHOQOL questionnaire goes beyond the traditional measures of symptom levels [40].

### Limitations of the study

The primary limitation of the present study was the small sample size of longitudinal respondents; only 49% completed all assessments over the 12 months. This is an unfortunate but common finding in longitudinal studies of injured and assaulted victims'. Other studies show high levels of dropouts with rates between 40% and 53% of the participants dropping out between the first and last assessment [35,45-50]. Attrition introduces questions about who is dropping out, whether the most or least symptomatic participants are not responding to all tree assessments. Such a bias would be a potentially serious methodological problem. However, in the present sample, respondents were comparable to dropouts in most ways except they tended to be older with somewhat higher education (table 1). The gender distribution was typical of people reporting violent crime (other than domestic assault) in Norway, but the age distribution was somewhat skewed with higher average age.(31], most likely explained by our participant's minimum age of 18 years. Further, there were no statistically significant differences between respondents and dropouts with regard to mean values on scales and subscales of IES-15 and WHOQOL-Bref. Future trauma research should consider whether the healthiest members of the sample usually respond to follow-ups in longitudinal studies [51].

Another limitation of the current study is that only 17% (12) of the longitudinal sample were females. The presence of female victims at T1 was 28 (20%). Our sample including few female victims are in accordance with another study focusing on the same kind of violence [47]. Additional research is needed to determine the degree to which our results would generalize to female victims of non-domestic violent assault.

The interview data in our study did not include clinical diagnostic interview such as the Clinician Administrated Posttraumatic Stress Scale (CAPS). Using only self-report questionnaires, to diagnose probable PTSD is another limitation. Nevertheless, in an attempt to reduce the latter point, we used two scales to assess PTSD symptoms, the Post-Traumatic Symptom Scale-10 (PTSS-10) and IES-15 which both have mainly used cut of scores to examine the severity of PTSD symptoms [3]. Specifically, presenting the cross-sectional analysis of 138 of the participants at T1 we found a similar occurrence of probable acute PTSD cases by using PTSS-10 and IES-15 [3]. We found a similar occurrence of probable PTSD cases by IES-15 and PTSS-10, but some differences concerning risk level cases. Same similarity was found in longitudinal analyses referred to in another paper [32]. IES-15 is examined in a study among crime victims by Wolfarth et al (2003), and found to be highly accurate in identifying PTSD cases, whether using DSM-IV or ICD-10 criteria. The questionnaire is screening for PTSD cases with high sensitivity (ranging between 0.93 and 1.00) and specificity (ranging between 0.78 and 0.84) [29].

## Conclusion

According to the present study, individuals diagnosed with full or partial symptoms of PTSD have a poor QoL compared with not diagnosed or normal populations. These QoL results demonstrate chronic, highly negative influences on the individual's perceived reality of their own situation. Early identification of probable PTSD and impact on QoL are very important because those who remain ill one year after the event rarely recover completely [7]. The present findings have clear practical implications. Firstly, clinical implications must be to prioritize interventions preventing development of PTSD, and secondly to follow up those with PTSD. In addition, in order to evaluate medical disability for financial compensation of victims of non-domestic violence, an assessment of QoL may be very useful.

PTSD has high impact on QoL in non-domestic victims of violence, as measured by the WHOQOL-Bref in all domains. The presence of PTSD in both the acute and later stages is a predictor of poor QoL. Such knowledge might provide guidance about how to effectively implement preventive and early intervention strategies in this group of victims. The individual's perception of his/her own life, in addition to the symptoms and the illness may increase both the patient's and the therapist's priority and effort as regards treatment. The diagnosis and symptoms may not be the most central concern of the patient, and use of QoL assessment puts the individual at the centre of inquiry. A more comprehensive approach by focusing on perceived QoL as well as symptom reduction as therapeutic strategies on PTSD patients, should consider advancing treatment outcome.

## Competing interests

The author(s) declare that they have no competing interests.

## Authors' contributions

VAJ conceived and designed the study, collected the data, performed statistical analysis and drafted the manuscript. AKW, LW and BRH participated in the design and revised the manuscript critically. DEE conducted the statistical analyses and revised the manuscript critically. All authors read and approved the final manuscript.
